# Cathepsin G, a Neutrophil Protease, Induces Compact Cell-Cell Adhesion in MCF-7 Human Breast Cancer Cells

**DOI:** 10.1155/2009/850940

**Published:** 2009-11-10

**Authors:** Tomoya Kudo, Hideaki Kigoshi, Takashi Hagiwara, Takahisa Takino, Masatoshi Yamazaki, Satoru Yui

**Affiliations:** ^1^Faculty of Pharmaceutical Sciences, Teikyo University, 1091-1 Sagamiko, Sagamihara, Kanagawa 229-0195, Japan; ^2^Department of Molecular Virology and Oncology, Cancer Research Institute, Kanazawa University, 13-1 Takara-machi, Kanazawa 920-0934, Japan

## Abstract

Cathepsin G is a serine protease secreted by activated neutrophils that play a role in the inflammatory response. Because neutrophils are known to be invading leukocytes in various tumors, their products may influence the characteristics of tumor cells such as the growth state, motility, and the adhesiveness between cells or the extracellular matrix. Here, we demonstrate that cathepsin G induces cell-cell adhesion of MCF-7 human breast cancer cells resulting from the contact inhibition of cell movement on fibronectin but not on type IV collagen. Cathepsin G subsequently induced cell condensation, a very compact cell colony, resulting due to the increased strength of E-cadherin-mediated cell-cell adhesion. Cathepsin G action is protease activity-dependent and was inhibited by the presence of serine protease inhibitors. Cathepsin G promotes E-cadherin/catenin complex formation and Rap1 activation in MCF-7 cells, which reportedly regulates E-cadherin-based cell-cell junctions. Cathepsin G also promotes E-cadherin/protein kinase D1 (PKD1) complex formation, and Go6976, the selective PKD1 inhibitor, suppressed the cathepsin G-induced cell condensation. Our findings provide the first evidence that cathepsin G regulates E-cadherin function, suggesting that cathepsin G has a novel modulatory role against tumor cell-cell adhesion.

## 1. Introduction

Cathepsin G is a 26-kDa neutral serine protease found in the azurophil granules of neutrophils and a subset of monocytes [1–3]. Human cathepsin G is synthesized as a 255-amino acid residue protein, including an 18-residue signal peptide and a 2-residue activation peptide at the N-terminus [4]. Cathepsin G, a major serine protease released by activated neutrophils, has been proposed to play an important role in inflammation through hydrolysis of a host of proteins, including chemoattractants, extracellular matrix (ECM), and hormonal factors [5]. In addition, the antibacterial action of cathepsin G and other azurophil granule proteins is thought to contribute significantly to the nonoxidative antibacterial capacity of neutrophils [6]. We previously observed that cathepsin G induces multicellular spheroids of mammary tumor cells [7]. Neutrophils are known to invade many tumor tissues and influence tumor development [8, 9]. However, the regulatory role of neutrophil proteases including cathepsin G in tumor progression and metastasis is not fully understood.

Cell-cell adhesion is critical for the normal development of multicellular organisms, tissue regeneration, immunological responses, and tumor metastasis [10]. Members of the cadherin superfamily of Ca^2+^-dependent cell-cell adhesion proteins are expressed in most organs and tissues of vertebrates and invertebrates [10–13]. Cadherin-mediated cell adhesion requires intracellular attachment of cadherin to the actin cytoskeleton [14–17]. Cadherins associate with the cytoskeleton through cytoplasmic interactions with catenins: *α*-catenin, *β*-catenin, and plakoglobin [16–18]. *α*-Catenin links E-cadherin to the actin cytoskeleton [19, 20] via its association with either plakoglobin or *β*-catenin [21, 22]. Cell-cell adhesion is initiated by weak binding between extracellular domains of E-cadherin that are present in a highly mobile pool at the plasma membrane. Subsequently, 2 cells achieve maximum contact, a process referred to as compaction. When more than 2 cells form contacts, E-cadherin and actin cables continue to reorganize in the process of cell condensation to form a more compact cell colony [23].

In the present study, we demonstrate that cathepsin G induces contact inhibition of cell movement and cell condensation of MCF-7 human breast cancer cells on fibronectin but not on type IV collagen. MCF-7 cells are known to use E-cadherin as the primary cell-cell adhesion molecule. Because the presented data indicate that cathepsin G induces the tight E-cadherin-mediated cell-cell adhesion in MCF-7 cells, we explored the molecular mechanism underlying the induction of cell condensation. The understanding of the reactions involved in the induction of cell-cell adhesion by cathepsin G might provide novel insights into tumor growth and metastasis.

## 2. Materials and Methods

### 2.1. Reagents

Cathepsin G purified from human neutrophils was purchased from Elastin Products (Owensville, MO). One unit is defined as the cathepsin G activity that releases 1 *μ*mole *p*-nitroanilide per minute from *N*-succinyl-Ala-Ala-Pro-Phe *p*-nitroanilide at pH 7.5 at 37°C. *α*_1_-Antitrypsin was obtained from Sigma-Aldrich (St. Louis, MO). Chymostatin, Go6976, GGTI-298, bisindolylmaleimide V, 8-Br-cGMP, and protein kinase G I*α* inhibitor were from Calbiochem (San Diego, CA). LY83583 was from Wako Pure Chemical Industries (Osaka, Japan). The immunological reagents used were anti-*α*-catenin (1G5), anti-*β*-catenin (E-5), anti-E-cadherin (67A4 and G-10), anti-Rap1 (5G7), anti-PKC*μ*/PKD1(C-20), and HRP-conjugated antimouse IgG_1 _antibodies (Santa Cruz Biotechnology, Santa Cruz, CA). The SCADS inhibitor kit I and II, consisting of 171 chemical inhibitors with about 140 different targets, was kindly provided by the Screening Committee of Anticancer Drugs (The Ministry of Education, Culture, Sports, Science and Technology, Japan).

### 2.2. Preparation of Extracellular Matrix Protein-Coated Dishes

The dishes (35 mm diameter; AGC Techno Glass, Chiba, Japan) were coated with 300 *μ*g/mL type IV collagen (Nitta Gelatin, Osaka, Japan), dried on a clean bench at room temperature, and washed 3 times with serum-free medium. Alternatively, the dishes were coated with 10 *μ*g/mL fibronectin or laminin (AGC Techno Glass) at 4°C overnight, blocked in 5% bovine serum albumin/phosphate-buffered saline (PBS) at room temperature for 1 hour, and washed once with serum-free medium.

### 2.3. Cell Culture and Induction of Cell Condensation

MCF-7 human breast cancer cells and BALB-MC.E12 mouse mammary tumor cells were maintained in RPMI1640 medium (Sigma-Aldrich) supplemented with 10% heat-inactivated fetal bovine serum (FBS; MP Biomedicals, Aurora, OH) and 80 *μ*g/mL of kanamycin (MP Biomedicals, LLC, Solon, OH) as previously described [7]. Cells were incubated at 37°C in a humidified atmosphere of 5% CO_2_. For analysis of induction of cell condensation, 1 × 10^5^ cells were cultured in dishes coated with type IV collagen or fibronectin. The seeding density was 70% in 5% FBS-containing medium estimated after 24 hours. On the other hand, for the observation of cell motility, 5 × 10^4^ cells were seeded at lower density (30%) to feasibly chase the cell movement. After washing, adherent cells were incubated in serum-free medium with 0.5 mU/mL cathepsin G for an additional 24 hours to induce cell condensation. Morphological observations of cultured MCF-7 cells were made by inverted, phase-contrast microscopy (ECLIPSE TE2000-U; Nikon, Tokyo, Japan). Original magnification ×200.

### 2.4. MTT Assay

Cathepsin G cytotoxicity toward the MCF-7 cells was evaluated by the 3-(4, 5 dimethyl-2-thiazolyl)-2, 5-diphenyl-2H-tetrazolium bromide (MTT) assay. Cells were cultured in 96-well microplates (AGC Techno Glass) coated with type IV collagen or fibronectin at 1 × 10^4^ cells/well. In the MTT assay, after the indicated periods, 10 *μ*L of MTT (Dojindo Laboratories, Kumamoto, Japan) (5 mg/mL) was added to each well, and the plates were incubated for an additional 3 hours. Next 50 *μ*L of the supernatant was then discarded, 100 *μ*L acid-isopropanol solution (0.04 N HCl in 2-propanol) was added to each well, and the optical density (595 nm) was measured with a microplate reader (Mutiscan MS-UV; Labsystems, Basingstoke, UK).

### 2.5. Disruption of E-Cadherin-Mediated Cell-Cell Adhesion

The cells were cultured in dishes (35 mm diameter) coated with fibronectin at 1 × 10^5^ cells/dish. Seeding efficiency was 50% or more in 5% FBS-containing medium estimated after 24 hours. After washing, adherent cells were cultured in serum-free medium with 0.5 mU/mL cathepsin G for 24 hours. These condensed cells were then incubated in serum-free medium supplemented with 400 *μ*M ethylene glycol-bis-(*β*-amino-ethyl ether) *N*, *N*, *N*′, *N*′-tetra-acetic acid (EGTA) (Nacalai Tesque, Kyoto, Japan) for 6 hours or 100 *μ*g/mL anti-E-cadherin antibody (HECD-1; Calbiochem) for 24 hours.

### 2.6. Immunoprecipitation and Western Blot Analysis

The cells were lysed in lysis buffer containing 25 mM Tris-HCl (pH 7.4), 150 mM NaCl, 1% Triton X-100, and protease inhibitor cocktail (Roche Diagnostics, Mannheim, Germany). After 10 minutes centrifugation at 15 000 × g, the insoluble pellet was removed, and the soluble extract was processed for immunoprecipitation. The soluble fraction was immunoprecipitated for 1 hour at 4°C. Immunocomplexes were adsorbed on Protein G Sepharose 4 Fast Flow (GE Healthcare, Amersham Place, UK). The samples were separated by sodium dodecyl sulfate-polyacrylamide gel electrophoresis (SDS-PAGE) on 8% gels and transferred onto polyvinylidene difluoride membranes (GE Healthcare). Membranes were blocked by incubation in Tris-buffered saline containing 0.2% Tween 20 (TBS-T) and 5% membrane blocking agent (GE Healthcare), followed by incubation for 1 hour with the indicated antibody. After extensive washing with TBS-T, membranes were incubated with secondary antibodies for 30 minutes at room temperature. After extensive washing with TBS-T, the blots were developed by incubation with a chemiluminescence substrate (GE Healthcare) and exposed to Hyperfilm ECL (GE Healthcare).

### 2.7. Rap1 Activity Assay

Rap 1 activation was examined in the cell lysates with Active Rap1 Pull-Down and Detection Kit (Pierce Biotechnology, Rockford, IL). In general, 1 × 10^5^ cells were cultured in dishes coated with fibronectin. Seeding efficiency was 70% in 5% FBS-containing medium estimated after 24 hours. After washing, adherent cells were incubated in serum-free medium with 0.5 mU/mL cathepsin G for 0–6 hours. Cells were washed once with ice-cold 1X PBS and lysed in ice-cold cell lysis buffer for 3 minutes on ice. Lysates were centrifuged at 16 000 × g for 15 minutes at 4°C, and total protein concentration in the supernatants was determined using the Bio-Rad Protein Assay. Active Rap1 pull-down assay was carried out with Active Rap1 Pull-Down and Detection Kit using supernatant aliquots.

### 2.8. Measurement of Intracellular cGMP Concentration

The cGMP concentrations in cell lysates were determined with the Direct cGMP Assay Kit (Assay Designs, Ann Arbor, MI). In general, 1 × 10^6^ cells were cultured on dishes coated with fibronectin in 5% FBS-containing medium for 24 hours. After washing, adherent cells were incubated in serum-free medium with 0.5 mU/mL cathepsin G for 0–3 hours. Cells were washed once with ice-cold 1X PBS and lysed in 0.1 M HCl for 20 minutes at room temperature. Lysates were centrifuged at 600 × g for 5 minutes to pellet the cellular debris, and the cGMP content of the supernatant was determined with the Direct cGMP Assay Kit.

## 3. Results

### 3.1. Cathepsin G Induces Contact Inhibition of Cell Movement and Cell Condensation in MCF-7 Cells

**Figure 1:**
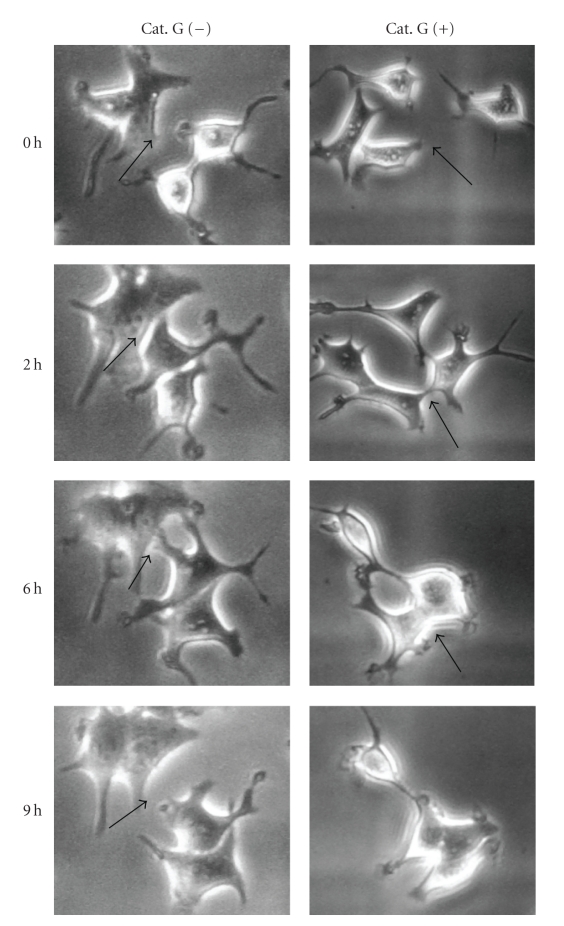
Cathepsin G induces contact inhibition of cell movement. 5 × 10^4^ MCF-7 cells were cultured in dishes coated with fibronectin for 24 hours. After washing, adherent cells were incubated in serum-free medium without or with 0.5 mU/mL cathepsin G. Cathepsin G-treated MCF-7 cells were analyzed by phase-contrast microscopy at the same locations in the indicated periods. Cathepsin G-induced contact inhibition of cell movement was observed at the original magnification: ×200. Arrows indicate adhesion sites between cells.

MCF-7 human breast cancer cells usually show temporal adhesion to each other and they repeat adhesion-dissociation cycles in vitro. When MCF-7 cells on fibronectin at the subconfluent condition were treated with cathepsin G, moving MCF-7 cells formed contacts with each other, thus promoting the formation of adherens junctions and maintaining cell-cell adhesion to prevent detachment from one other (Figure 1). This phenomenon has been known as “contact inhibition of cell movement” [24].

**Figure 2:**
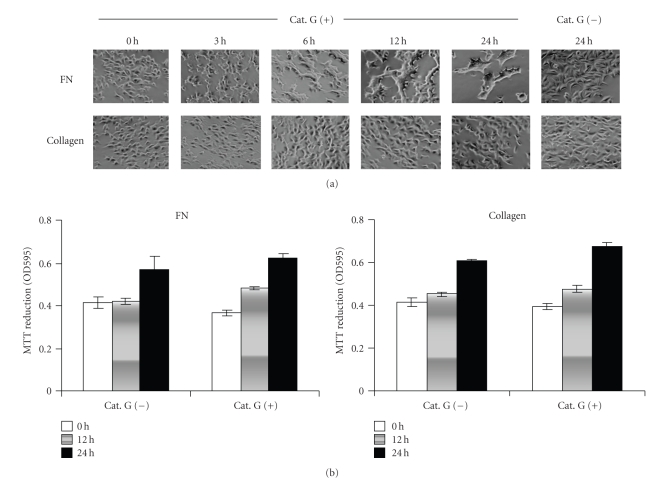
Cathepsin G induces cell condensation in MCF-7 cells on fibronectin, but not on type IV collagen. 1 × 10^5^ MCF-7 cells were cultured in dishes coated with fibronectin or type IV collagen for 24 hours. After washing, adherent cells were incubated in serum-free medium without or with 0.5 mU/mL cathepsin G. (a) Cathepsin G-induced cell condensation of MCF-7 cells was analyzed by phase-contrast microscopy in the indicated periods as described in Materials and Methods. (b) MTT-reducing activity was measured at the indicated times. Bars represent the standard deviation. Analyses were performed in triplicate in duplicate experiments.

Next, we observed changes in cell morphology under more confluent conditions (Figure 2(a)). The incubation of MCF-7 cells without cathepsin G for 24 hours did not result in a change in their morphology on fibronectin. On the other hand, when MCF-7 cells were treated with cathepsin G under the same condition, cell condensation of MCF-7 was induced in a stepwise manner: single cells initially form chains within 3 hours and then aggregate into loose, irregular clumps of cells with smooth margins between 6 and 24 hours.

In the breast tissue, tumor cells were surrounded with various components of extracellular matrix, such as fibronectin, laminin, and collagens, the components of which varied during tumor progression [25]. Accordingly, we examined the effect of extracellular matrix components as culture substrates on the cell condensation-inducing activity of cathepsin G. We observed that cathepsin G also induces cell condensation on laminin (data not shown) but not on type IV collagen (Figure 2(a)). To exclude the possibility that the varying culture substrate outcomes are due to the loss of viability of MCF-7 cells, the viability was evaluated by MTT assay. As shown in Figure 2(b), MCF-7 cells remained viable with evidence of growth up to 24 hours both on fibronectin and type IV collagen.

### 3.2. Cathepsin G Promotes E-Cadherin/Catenin Complex Formation in MCF-7 Cells

**Figure 3:**
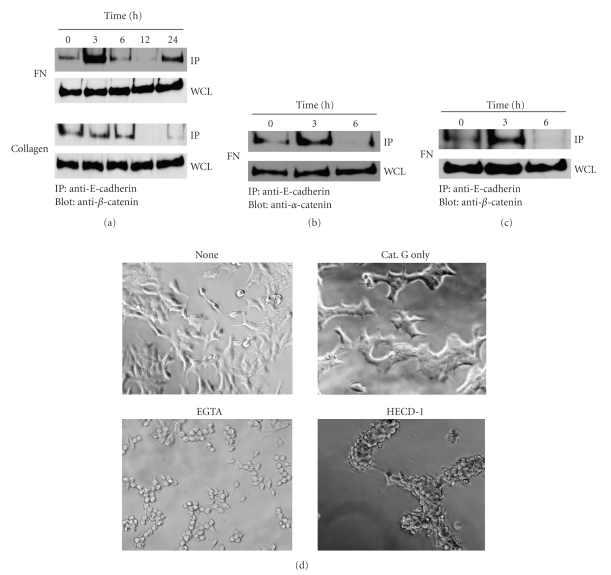
Cathepsin G-induced E-cadherin/catenin complex formation and E-cadherin-mediated cell-cell adhesion of MCF-7 cells. MCF-7 cells were cultured in dishes coated with fibronectin for 24 hours. After washing, adherent cells were incubated in serum-free medium without or with 0.5 mU/mL cathepsin G. At each indicated culture time, the cells were lysed, and E-cadherin/catenin complex formation of MCF-7 cells was analyzed by immunoprecipitation and western blot analysis as described in Materials and Methods. (a) and (b) Immunocomplexes with anti-E-cadherin were analyzed by immunoblotting using an anti-*β*-catenin (a) or anti-*α*-catenin antibody (b). Whole-cell lysates (WCLs) were immunoblotted with an anti-*β*-catenin (a) or anti-*α*-catenin antibody (b). (c) BALB-MC.E12 mouse mammary tumor cells were analyzed as shown in (a). (d) Treatments inhibiting E-cadherin-mediated cell-cell adhesion disrupt cathepsin G-induced cell condensation. MCF-7 cells were cultured in 5% FBS-containing medium on fibronectin for 24 hours. After washing, cell condensation was induced by cathepsin G for 24 hours. Condensed cells were then subjected to serum-free medium supplemented with 400 *μ*M EGTA for 6 hours or HECD-1 (100 *μ*g/mL) for 24 hours and then analyzed by phase-contrast microscopy. Cathepsin G-induced cell condensation was analyzed at the original magnification: ×200.

Cell condensation is induced by the increased strength of E-cadherin-mediated cell-cell adhesion [23]. We next analyzed E-cadherin/catenin complex formation in the time course of cathepsin G treatment. E-cadherin/*β*-catenin complexes were transiently induced at 3 hours on fibronectin (Figure 3(a)). This result parallels the observation of phase-contrast microscopy in Figure 2(a). The complex formation disappeared at 6 hours and reappeared at 24 hours. Figure 3(b) shows that *α*-catenin also associates with this complex at 3 hours. In contrast, the E-cadherin/cytoskeleton association was not induced on type IV collagen at any time period. This result again parallels the observation of Figure 2(a).

To know whether the transient pattern of E-cadherin/catenin complex formation induced by cathepsin G is restricted to MCF-7 cells, we examined complex formation in BALB-MC.E12 mouse mammary tumor cells. As shown in Figure 3(c), cathepsin G also stimulated the transient E-cadherin/*β*-catenin complex formation at 3 hours in the mouse tumor cells, raising the possibility that the transient formation of the complex is a common event in cathepsin G-induced cell condensation.

E-cadherin-mediated cell-cell adhesion is known to be disrupted by a well-established Ca^2+^ switch procedure, which involves the removal of extracellular Ca^2+^ with the specific chelator EGTA [26]. Thus, to study the critical role of E-cadherin in cathepsin G-induced cell condensation, we examined whether EGTA or HECD-1, a neutralizing antibody against E-cadherin, affects the state of cathepsin G-induced cell condensation. As shown in Figure 3(d), the tight cell condensation disappeared after EGTA or HECD-1 treatment. The results support that cell condensation induced by cathepsin G was mediated by E-cadherin. Although cell morphology did not return to the adherent morphology after use of these agents, the adherent capacity of the cells against the culture substrate might be also influenced by cathepsin G.

**Figure 4:**
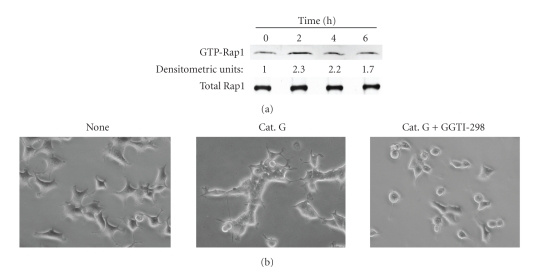
Rap1 activation is involved in induction of cell condensation by cathepsin G. (a) MCF-7 cells were cultured in dishes coated with fibronectin. After 24 hours, the adherent cells were incubated in serum-free medium with 0.5 mU/mL cathepsin G for the indicated periods. Active Rap1 was analyzed by the pull-down assay. Western blotting of whole cell lysates was used to assess total levels of Rap1 (Total Rap1). The densitometric units represent Rap1 activity relative to cathepsin G-untreated cells (0 hour) taken as 1.0. (b) The adherent MCF-7 cells were treated with cathepsin G in the absence or presence of a Rap1 inhibitor GGTI-298 (5 *μ*M) for 5 hours. Cells were then analyzed by phase-contrast microscopy original magnification: ×200.

Rap1 reportedly plays a key role in the formation of cadherin-based cell-cell junctions [27]. Rap1 guanine nucleotide exchange factors such as C3G and PDZ-GEF are directly linked to E-cadherin [28]. To explore the molecular mechanism underlying cathepsin G-promoted E-cadherin-mediated cell-cell adhesion, we determined Rap1 activity (Figure 4(a)). Rap1 activation was evident in cells treated with cathepsin G for 2–4 hours as compared to cathepsin G-untreated cells. This result parallels the cathepsin G-promoted E-cadherin/catenin complex formation of Figure 2(a), suggesting that Rap1 regulates cathepsin G-promoted E-cadherin-mediated cell-cell adhesion. To further examine whether Rap1 participates in cathepsin G-induced cell condensation, we determined the effect of GGTI-298, an inhibitor of geranylgeranylation of GTPases such as Rap1 [29]. As shown in Figure 4(b), GGTI-298 completely inhibited induction of cell condensation.

**Figure 5:**
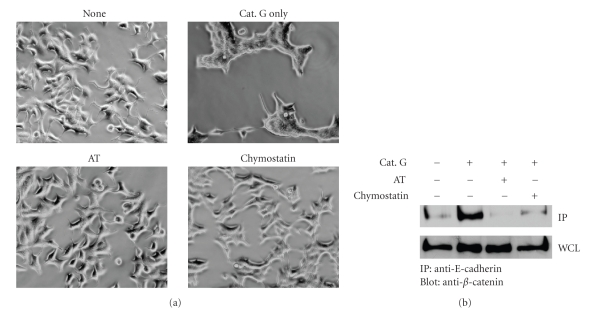
Enzymatic activity of cathepsin G is required for cathepsin G-promoted E-cadherin-mediated cell-cell adhesion. MCF-7 cells were incubated in 5% FBS-containing medium on fibronectin for 24 hours. (a) After washing, the adherent cells were treated with cathepsin G in the absence or presence of 50 *μ*g/mL chymostatin or 200 *μ*g/mL *α*_1_-antitrypsin (AT) for 24 hours. Cathepsin G-induced cell condensation was observed by phase-contrast microscopy. (b) After washing, adherent cells were treated with cathepsin G in the absence or presence of 50 *μ*g/mL chymostatin or 200 *μ*g/mL *α*_1_-antitrypsin (AT) for 3 hours. The immunocomplexes with anti-E-cadherin were then analyzed by immunoblotting with anti-E-cadherin using an anti-*β*-catenin antibody.

To analyze whether enzymatic activity of cathepsin G is required in cathepsin G-induced cell condensation, we used the serine protease inhibitors chymostatin and *α*_1_-antitrypsin. In phase contrast microscopy, although the morphology of MCF-7 cells treated with cathepsin G changed to that of cells during cell condensation, MCF-7 cells incubated with cathepsin G in the presence of chymostatin or *α*_1_-antitrypsin did not show morphological changes (Figure 5(a)). Moreover, chymostatin or *α*_1_-antitrypsin inhibited cathepsin G-promoted E-cadherin/*β*-catenin complex formation at 3 hours (Figure 5(b)). These results indicate that the enzymatic activity of cathepsin G is required for cathepsin G-promoted E-cadherin-mediated cell-cell adhesion.

### 3.3. Cathepsin G Increases PKD1/E-Cadherin Complexes in MCF-7 Cells

**Figure 6:**
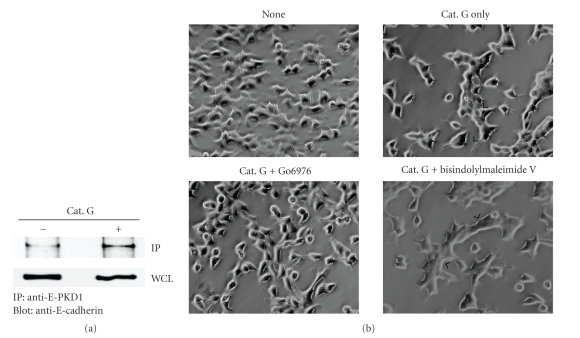
Cathepsin G promotes E-cadherin/PKD1 complex formation in MCF-7 cells. MCF-7 cells treated with cathepsin G for 10 hours were analyzed by immunoprecipitation and western blot analysis as described in Materials and Methods. (a) Immunocomplexes with anti-PKD1 antibody or whole-cell lysates (WCLs) were analyzed by immunoblotting using an anti-E-cadherin antibody. (b) After washing, the adherent cells were treated with cathepsin G in the absence or presence of 5 *μ*M Go6976 or 5 *μ*M of the negative control compound bisindolylmaleimide V for 5 hours. Cells were then analyzed by phase-contrast microscopy. Cathepsin G-induced cell condensation was analyzed at the original magnification: ×200. Arrowheads indicate the site of cell condensation in the cathepsin G-treated cells, while cell condensation was not observed in the cells treated with cathepsin G and Go6976.

The cadherin/catenin complex of proteins is a major target of posttranslational modifications such as phosphorylation and dephosphorylation. Recent data show that E-cadherin is phosphorylated by PKD1, and increased kinase activity and overexpression of PKD1 increases cell aggregation and decreases cell motility [30]. Therefore, we next studied the role of PKD1 in cell condensation induced by cathepsin G. Figure 6(a) shows cathepsin G-promoted E-cadherin/PKD1 complex formation in MCF-7 cells. To determine whether PKD1 activity is involved in the cell condensation signaling pathway, we examined the effect of the selective inhibitor Go6976 of PKD1 [31]. As shown in Figure 6(b), the cathepsin G-induced cell condensation was obviously decreased in the presence of Go6976. On the other hand, bisindolylmaleimide V, the negative control compound of Go6976 [32], did not affect the induction of cell condensation. These data indicate that PKD1 plays an important role in the signaling of cathepsin G-promoted E-cadherin-mediated cell-cell adhesion.

### 3.4. LY83583 Inhibits Cathepsin G-Promoted E-Cadherin-Mediated Cell-Cell Adhesion

**Figure 7:**
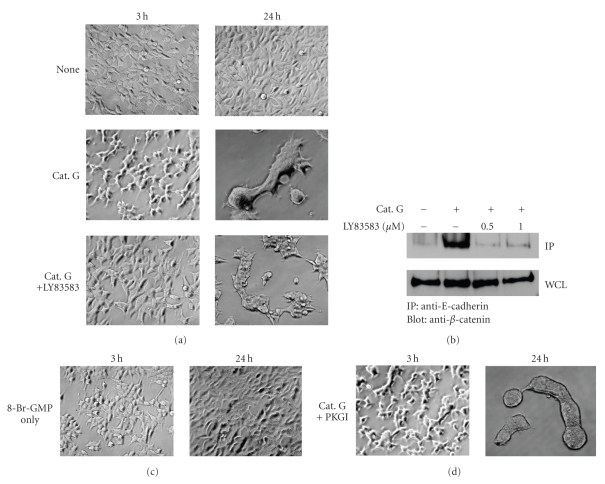
Guanylyl cyclase inhibitor LY83583 has an inhibitory effect on cathepsin G-promoted E-cadherin-mediated cell-cell adhesion. MCF-7 cells were incubated in 5% FBS-containing medium on fibronectin for 24 hours. Cathepsin G-induced cell condensation was analyzed at the original magnification: ×200. (a) After washing, adherent cells were treated with cathepsin G in the absence or presence of 1 *μ*M LY83583 for 24 hours. Cells were then analyzed by phase-contrast microscopy at 3 hours and 24 hours. (b) After washing, adherent cells were treated with cathepsin G in the absence or presence of LY83583 for 3 hours. The immunocomplexes were then analyzed by immunoblotting using an anti-*β*-catenin antibody. (c) Adherent cells were treated with 1 *μ*M 8-Br-cGMP, and cells were then analyzed by phase-contrast microscopy at 3 hours and 24 hours. (d) After washing, adherent cells were treated with cathepsin G in the absence or presence of 1 *μ*M PKGI for 24 hours. Cells were then analyzed by phase-contrast microscopy at 3 hours and 24 hours.

As described earlier, we obtained evidences suggesting that Rap1 and PKD1 may be involved in the signaling pathway of cathepsin G-induced cell condensation. However, other members, especially those involved in the upstream events of the signaling pathway, remain unknown. To clarify cathepsin G-induced signal transduction, which is associated with the increase in the strength of E-cadherin-mediated cell-cell adhesion, the effects of various chemical inhibitors were examined. To achieve this, we used the SCADS inhibitor kit I and II, consisting of 171 chemical inhibitors with about 140 different targets provided by the Screening Committee of Anticancer Drugs in Japan. Among these inhibitors, guanylyl cyclase inhibitor LY83583 markedly inhibited the early phase of cathepsin G-induced MCF-7 cell condensation. As shown in Figure 7(a), cathepsin G alone induced the morphology of cell chains in the early phase of cell condensation. In the presence of LY83583, it did not bring about such morphological changes. Furthermore, tight cell condensation was not observed in the presence of LY83583 as compared to treatment with cathepsin G only at 24 hours. Similarly, LY83583 inhibited cathepsin G-promoted E-cadherin/*β*-catenin complex formation (Figure 7(b)).

We next studied whether cGMP signaling involves cathepsin G-promoted E-cadherin-mediated cell-cell adhesion. However, our results indicated that there is not involvement of cGMP signaling; cathepsin G did not elevate the intracellular cGMP concentration in MCF-7 cells (not shown), and cell condensation was not induced by 8-br-cGMP (Figure 7(c)), a membrane-permeable and phosphodiesterase-resistant analogue of cGMP. Moreover, induction of cell condensation by cathepsin G was not inhibited by protein kinase G I*α* inhibitor (PKGI) (Figure 7(d)). These results suggest that LY83583 inhibits cathepsin G-induced cell condensation by a mechanism, which is irrelevant to the cGMP-PKG pathway. It is important to elucidate the mode of action of LY83583 on the signal transduction cascade in future research.

## 4. Discussion

Cathepsin G, a major serine protease released by activated neutrophils, has been proposed to play an important role in tissue remodeling at sites of tissue injury [5, 33, 34]. In addition, it is generally accepted that neutrophils often exist in tumors and influence tumor development [8, 9, 35]. Nevertheless, the role of neutrophils in preventing tumor development remains largely unexplained at the molecular level. Here, we show that contact inhibition of cell movement and cell condensation is induced by cathepsin G in MCF-7 human breast cancer cells. However, cathepsin G-induced cell condensation was observed in cultures in which fibronectin or laminin was used as culture substrates but not in those in which type IV collagen was used. It is unclear why cathepsin G-induced cell condensation is influenced by the type of ECM protein used. We are designing experiments to study the possibility that collagen-dependent cell adhesion affects the cells via integrin-mediated outside-in signaling.

It has been reported that cadherin-mediated cell adhesion requires the intracellular attachment of cadherin to the actin cytoskeleton [14–17] and that cadherins associate with the cytoskeleton through cytoplasmic interactions with the catenins *α*-catenin, *β*-catenin, and plakoglobin [16–18]. We elucidated that cathepsin G markedly induced E-cadherin/catenin complex formation on fibronectin but not on type IV collagen. Interestingly, the E-cadherin/cytoskeleton association was transient; it occurred at the earlier phase of cell condensation at 3 hours, disappeared after 6 hours, and reappeared at 24 hours. These results indicate that E-cadherin possibly accesses the cell-cell contact interface and promotes the association with the cytoskeleton in the early phase of the reaction, and that once homotypic association of E-cadherin molecules is formed, the cytoskeleton is subsequently dissociated from E-cadherin. The formation of tight cell condensation at the later phase (24 hours) may probably require the E-cadherin/cytoskeleton association. When the tight cell condensation was treated by EGTA or HECD-1, the cell boundaries became evident. These results indicate that cathepsin G regulates E-cadherin function and increases the strength of E-cadherin-mediated cell-cell adhesion.

E-cadherin plays an important role in tumor metastasis. In some tumors, E-cadherin dysfunction occurs and the downregulation of E-cadherin is an important step in tumor cell invasion and metastasis [36]. It can be speculated by the data presented here that neutrophil-derived cathepsin G prevents tumor cell invasion by inducing tight cell-cell adhesion. On the contrary, E-cadherin-mediated collective migration reportedly promotes tumor cell invasion and metastasis [37–39]. In addition, the signal induced by E-cadherin-mediated cell adhesion replaces the integrin-mediated cell growth signal and prevents anoikis [40]. In these cases, cathepsin G-induced tumor cell adhesion via E-cadherin may exert deleterious effects on tumor development and metastasis. The in vivo effect of cathepsin G is thus an important subject of future study to learn the role of tumor-infiltrating neutrophils.

It has been suggested that Rap1 regulates cathepsin G-promoted E-cadherin-mediated cell-cell adhesion. In addition, promotion of E-cadherin/PKD1 complex formation is suggested to be required for cathepsin G-promoted E-cadherin-mediated cell-cell adhesion. Although the relationship between Rap1 and PKD1 has not yet been clarified, these factors may be key players in a novel signaling pathway in cathepsin G-induced signal transduction. The enzymatic activity of cathepsin G is required for the induction of cell condensation. Cathepsin G reportedly activates protease-activated receptor (PAR)-4 in platelets [41]. It is important to consider whether signaling through PAR is involved in this reaction. We also demonstrated that the guanylyl cyclase inhibitor LY83583 has an inhibitory effect on cathepsin G-promoted E-cadherin-mediated cell-cell adhesion. However, intracellular cGMP was not augmented by cathepsin G. In addition, cathepsin G-induced cell condensation was not induced by 8-br-cGMP and was not inhibited by PKGI. Although the mechanism of action of LY83583 is unknown at the present time, the compound might be a useful tool to uncover the signal transduction mechanism involved upstream of Rap1 and PKD1.

In summary, our results provide novel important insights into cathepsin G functions and indicate that cathepsin G increases the strength of E-cadherin-mediated cell-cell adhesion in MCF-7 cells, with important implications for tumor development and metastasis. We postulate that cathepsin G secreted by infiltrated neutrophils in tumor tissue may have a novel modulatory role in tumor development.
